# Genomic content of chemosensory receptors in two sister blister beetles facilitates characterization of chemosensory evolution

**DOI:** 10.1186/s12864-020-06974-4

**Published:** 2020-08-26

**Authors:** Yuan-Ming Wu, Yang-Yang Liu, Xiang-Sheng Chen

**Affiliations:** 1 School of Basic Medical Sciences, Guizhou Medical University, Guiyang, Guizhou P.R. China 550025; 2 Key Laboratory of Environmental Pollution Monitoring and Disease Control, Ministry of Education, Guizhou Medical University, Guiyang, Guizhou P.R. China 550025; 3 Institute of Entomology/Special Key Laboratory for Development and Utilization of Insect Resources, Guizhou University, Guiyang, Guizhou P.R. China 550025

**Keywords:** Blister beetle (*Spanishfly*), Chemosensory, Gustatory receptor, Odorant receptor, Ionotropic receptors

## Abstract

**Background:**

More than 2500 species belong to the Meloidae family (Coleoptera: Tenebrionoidea), members of which produce the potent defensive blistering agent cantharidin and are commonly known as blister beetles or *Spanishflies*. Cantharidin has recently been used for cancer therapy. *Hycleus cichorii* and *Hycleus phaleratus* have been used in traditional Chinese medicine for more than 2000 years due to their ability to biosynthesize cantharidin. To understand the role of the chemosensory system in beetle evolution, we comparatively analysed the chemosensory receptor families of both blister beetle species and compared them with those of other beetles.

**Results:**

We identified 89 odorant receptors (ORs), 86 gustatory receptors (GRs), and 45 ionotropic receptors (IRs) in *H. phaleratus* and 149 ORs, 102 GRs and 50 IRs in *H. cichorii*. Nine groups of beetle ORs were recovered, and a similar pattern of ORs in Coleoptera emerged. Two evident expanded clades in *Hycleus* (Groups 5A and 3) were reconstructed in the phylogenetic tree. Four of eight genes with evidence of positive selection were clustered in the expanded clades of Group 5A. Three, eight and three orthologous pairs of CO_2_, sugar and fructose receptors, respectively, were identified in both blister beetles. Two evident expanded clades of putative bitter GRs in *Hycleus* were also found, and the GR in one clade had notably low divergence. Interestingly, IR41a was specifically expanded in blister beetles compared to other insects identified to date, and IR75 was also clearly expanded in both blister beetles based on our phylogenetic tree analysis. Moreover, evidence of positive selection was detected for eight ORs, three GRs and two IRs, half of which were from five duplicate clades.

**Conclusions:**

We first annotated the chemosensory receptor families in a pair of sister beetle genomes (Meloidae: *Hycleus*), which facilitated evolutionary analysis of chemosensory receptors between sibling species in the Coleoptera group. Our analysis suggests that changes in chemosensory receptors have a possible role in chemical-based species evolution in blister beetles. Future studies should include more species to verify this correlation, which will help us understand the evolution of blister beetles.

## Background

To adapt to varied environments and ecological niches, insects rely upon their sensory system to distinguish chemical signals, such as pheromones and plant volatiles. An understanding of the relative changes in genes among closely related insect species is required to help elucidate the role of chemosensory adaptation in different environments because the chemical-sensing system is important for host and mate recognition [1]. The evolutionary events of gene duplication, amino acid mutation and gene expression variation may play essential roles during speciation or adaptive evolution. For example, during the process of host specialization of *Drosophila sechellia*, rapid evolution events and expression divergence were widely detected in chemosensory genes, such as gustatory receptor (GR) genes, odorant receptor (OR) genes and genes encoding odorant-binding proteins (OBPs) [2–4]; certain chemoreceptor genes with mutations might play key roles in local adaptation and reproductive isolation in the pea aphid *Acyrthosiphon pisum* [5]; in *Pyrrhalta* beetles, protein mutations and changes in the expression of some particular chemosensory genes are associated with reproductive isolation and host shift [6]; and the changes in the expression of genes encoding particular proteins (OBPs) and ORs are associated with the host plant shift in *Chrysomela lapponica* [7].

In insects, at least three chemosensory receptor families are involved in the recognition of chemical signals. OR gene families are usually expressed in olfactory sensory neurons and are involved in the detection of volatile chemicals [8–12]. GRs are mainly involved in the detection of contact chemicals or carbon dioxide [13–15], and ionotropic receptors (IRs) are known to recognize acids, aromatics and nitrogen-containing compounds [16, 17]. These genes allow insects to seek and select hosts and mates and thus can affect adaptive changes in closely related species.

More than 2500 species of insects commonly known as blister beetles or *Spanishflies* belong to the Meloidae family, and more than 1500 of these beetle species are known to produce cantharidin [18]. Cantharidin (C_10_H_12_O_4_) is widely used in anti-insect and bacterial products in agricultural and medical applications worldwide [18–20]. Recently, cantharidin and its derivatives have been used to treat several cancers, including stomach, liver, lung and oesophageal cancers [21–23]. The dried body of this beetle has been used as a traditional medicine in China for the past 2000 years. However, only *Hycleus cichorii* Linnaeus and *Hycleus phaleratus* Pallas are widely used and listed in the Pharmacopoeia of the People’s Republic of China [24].

These two beetles have largely overlapping sympatric ranges in China and a similar emergence phenology and appearance, except that *H. phaleratus* has a larger body size than *H. cichorii*. These beetles are mainly distributed in Southwest China and feed on the eggs of Locustidae species during the larval period and on legumes and cucurbitaceous crops in adulthood. However, in our field survey experience, the *H. phaleratus* population has declined faster than the *H. cichorii* population in recent years due to destruction of their habitats by human activity, including capture by humans. Therefore, an understanding of the changes that underlie speciation between these two sister species and comparison of the chemosensory proteins that are highly important for insect survival and reproduction and represent interesting strategies for species evolution can provide important insights. Although several studies have compared differences in chemosensory genes between sister species [3–7, 25, 26], most of these studies have investigated this aspect partly by RNA-seq, and relatively few studies have comprehensively examined how sister taxa differ in this aspect, especially in Coleoptera groups. In this study, we identified the members of three receptor families involved in chemosensory perception in a pair of sister blister beetles and analysed the evolution of these receptors in beetles.

## Results

The annotated candidate chemosensory receptors were identified by tBLASTn and manual verification. In total, we identified 89 ORs, 86 GRs and 45 IRs in *H. phaleratus* and 149 ORs, 102 GRs and 50 IRs in *H. cichorii* (Table 1, Additional file 1 and Additional file 2). Moreover, 12 and 14 ionotropic glutamate receptors (iGluRs; five full-length genes in each) were identified in *H. phaleratus* and *H. cichorii*, respectively (Table 1, Additional file 1 and Additional file 2). All the coding sequences (CDSs) and proteins identified in this study are listed in Additional file 1, and all the gene locations and exon boundaries are listed in Additional file 2.

**Table 1:** Number of Chemosensory Genes in the Genome of the Two Sister Blister Beetles *H. phaleratus* and *H. cichorii*. The numbers in parentheses are the gene numbers from the phylogenetic tree, the full-length genes and pseudogenes

Species	ORs	GRs	IRs/iGluRs
*H. phaleratus*	**89(72/44/20)**	**86(74/68/9)**	**45(45/25/6)/12(12/5/1)**
*H. cichorii*	**149(130/77/30)**	**102(87/62/19)**	**50(50/30/8)/14(14/5/1)**
Orthologous pairs [positively select]	**53 [8]**	**55 [3]**	**39 [2]/11 [1]**

### Odorant receptors

**Fig. 1:**
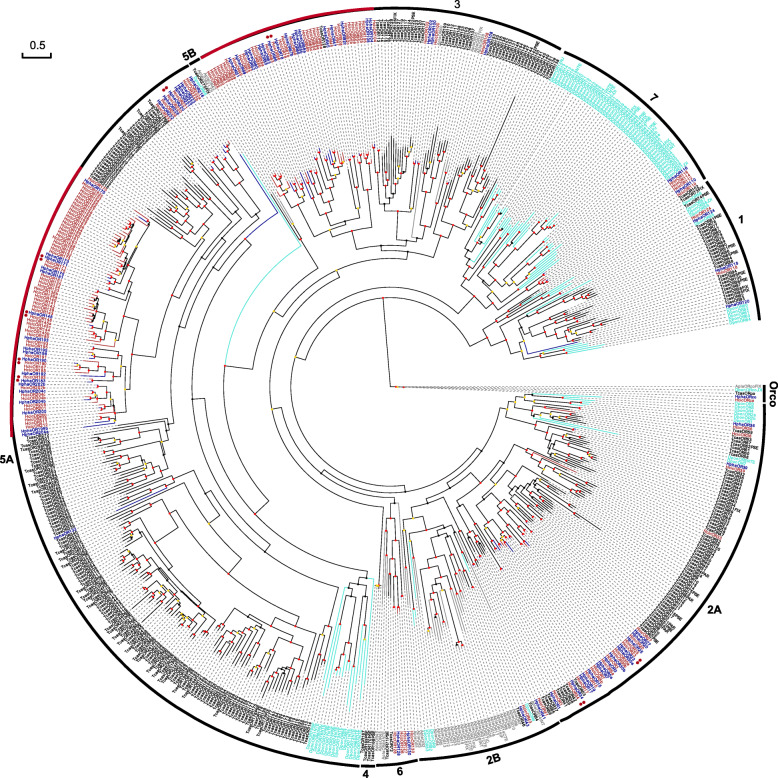
Maximum Likelihood Phylogeny Based on the Protein Sequences of the Candidate ORs. Red: *H. cichorii* (Hcic); blue: *H. phaleratus* (Hpha); black: *T. castaneum* (Tcas); turquoise: *D. ponderosae* (Dpon); and grey: *A. planipennis* (Apla). The tree is based on MAFFT alignment, was constructed using RAxML, and was rooted with the conserved lineage of Orco proteins. The coloured dots indicate nonparametric bootstrap support (%): red: ≥ 75, yellow: 50–74, and black: < 50. Two major *Hycleus* expanded clades are evident (red arc). The red asterisk (*) marks the positively selected gene pairs

We identified 89 (including 44 full-length and 20 pseudogenes) and 149 (including 77 full-length and 30 pseudogenes) ORs in *H. phaleratus* and *H. cichorii*, respectively. In both species, the numbers of introns in the full-length OR genes vary between two and six, with more than 90% ranging from three to five. Additionally, HcicORco and HphaORco were also identified (Fig. 1). Recently, the coleopteran OR subfamilies were reclassified by Mitchell et al. [27]. Based on Mitchell’s study, nine beetle OR groups were recovered in our data (Fig. 1). The blister beetle ORs are distributed in seven groups, lacking Group 4 and 5B, indicating loss of ORs from these two groups. Most *Hycleus* ORs appeared in Groups 5A, 3 and 2A (Fig. 1). Most ORs of *Dendroctonus ponderosae* were classified in Group 7, whereas the largest expansion in *Agrilus planipennis* was observed in Group 2B (Fig. 1), which is consistent with previous reports [28]. Notably, two evident expanded OR clades were identified based on our phylogenetic tree (Fig. 1, red arc in Groups 3 and 5A), containing more than 50% of the *Hycleus* ORs. Although up to 67% more ORs were identified in *H. cichorii* than in its sister species (149 vs 89 ORs), nearly all these additional *H. cichorii* ORs were clustered in two clades. The ORs in the other clades were almost all single-copy (1:1) orthologues in blister beetles. Moreover, we identified 53 pairs of orthologues between the two beetle species based on the maximum likelihood (ML) tree, where 8 pairs of orthologues showed evidence of positive selection (Table 1, Fig. 1 and Additional file 3). Among these pairs, a total of 5 pairs, 2 pairs and 1 pair were distributed in Groups 5A, 2A and 3, respectively. Interestingly, four pairs of genes with evidence of positive selection were clustered in the expanded clades of Group 5A (Fig. 1).

### Gustatory receptors

**Fig. 2:**
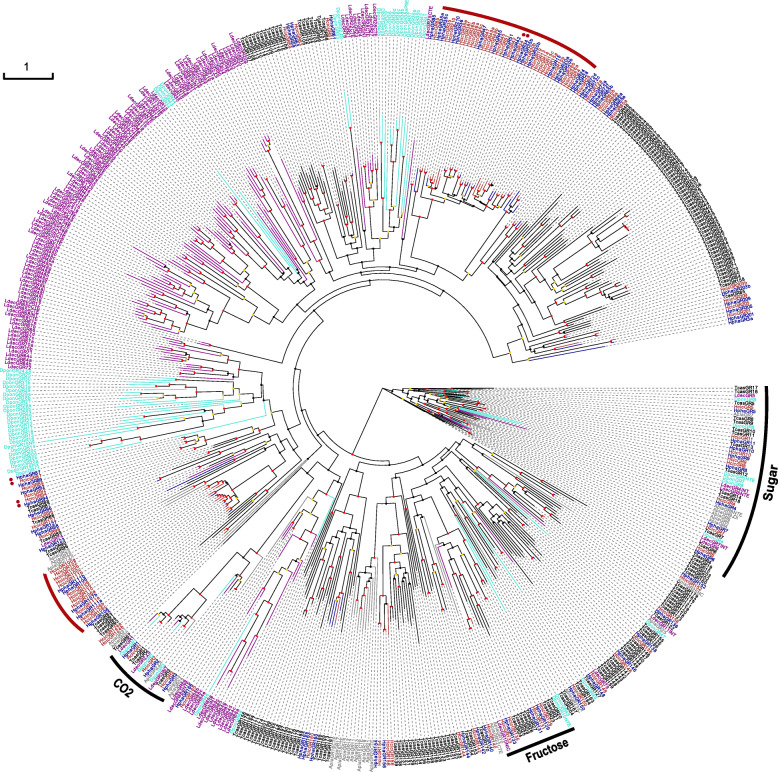
Maximum Likelihood Phylogeny Based on the Protein Sequences of the Candidate GRs. Red: *H. cichorii* (Hcic); blue: *H. phaleratus* (Hpha); black: *T. castaneum* (Tcas); turquoise: *D. ponderosae* (Dpon); purple: *L. decemlineata* (Ldec) and grey: *A. planipennis* (Apla). The tree is based on MAFFT alignment, was constructed using RAxML, and was rooted with the conserved lineage of putative sugar receptors. The coloured dots indicate nonparametric bootstrap support (%): red: ≥ 75, yellow: 50–74, and grey: < 50. Two recent *Hycleus* expanded clades are evident (red arc). The red asterisk (*) marks the positively selected gene pairs

We identified 86 (including 68 full-length genes and 9 pseudogenes) and 102 (including 62 full-length genes and 19 pseudogenes) GRs in *H. phaleratus* and *H. cichorii*, respectively. Three pairs of CO_2_ receptors were identified (named GR1–3), containing two introns, five introns and one intron, respectively, and this group presented 1:1 orthologues among these beetles in our phylogenetic tree (Fig. 2). Eight pairs of conserved sugar receptors were also detected in this study (named GR4–11; Fig. 2), containing four to six introns. Additionally, three pairs of homologues and an additional paralogue *H. cichorii* GR, which functions as a fructose receptor, were detected, containing two to five introns. The flour beetle was annotated with the most (eight) copies of the fructose receptor in these six analysed beetle species, followed by the blister beetles, and the remaining three beetles had only one copy. The remaining GRs are putative bitter GRs, and most full-length bitter GRs contain one or two introns (with a total range of one to five), similar to those in *D. ponderosae* and *A. planipennis* [28]. Almost all bitter GRs from the other four species were grouped into small to large species-specific expanded clades. Two blister beetle expanded clades emerged in our phylogenetic tree, which included ~ 44% bitter GRs (red arc in Fig. 2). Similar to the expanded clades of ORs, these GRs in one clade showed notably low divergence among each other. The bitter GRs in the *Hycleus* genus exhibit high conservation, and more than 55 pairs of orthologues were shared by the two blister beetle species, with more than 2/3 of the *H. phaleratus* GRs existing in orthologue pairs (Fig. 2). A total of 3 pairs of orthologues between the blister beetle species showed evidence of positive selection, and one pair (GR34) was located in an expanded clade (Table 1, Fig. 2 and Additional file 3).

### Ionotropic receptors

**Fig. 3:**
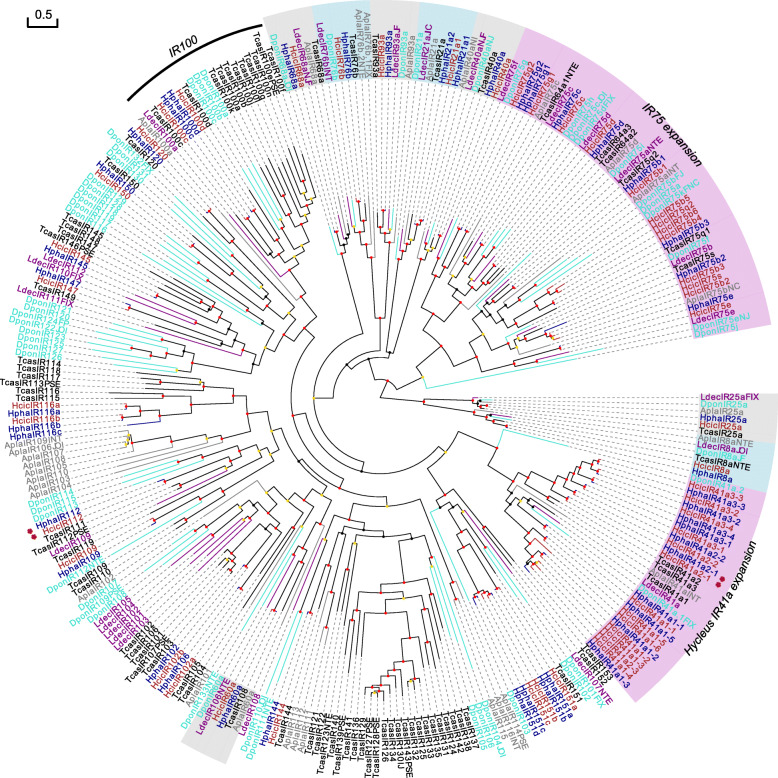
Maximum Likelihood Phylogeny Based on the Protein Sequences of the Candidate IRs and iGluRs. Red: *H. cichorii* (Hcic); blue: *H. phaleratus* (Hpha); black: *T. castaneum* (Tcas); turquoise: *D. ponderosae* (Dpon); purple: *L. decemlineata* (Ldec) and grey: *A. planipennis* (Apla). The tree is based on MAFFT alignment, was constructed using RAxML, and was rooted with the conserved lineage of putative IR8a/25a clade. The coloured dots indicate nonparametric bootstrap support (%): red: ≥ 80, yellow: 50–79, and black: < 50. The ‘antennal IRs’ are shaded with colours. Major *Hycleus*-specific expansion of ‘IR41a’ is evident, and the ‘IR75’ clade is expanded in both the *Hycleus* genus and bark beetle (plum shade). The red asterisk (*) marks the positively selected gene pairs

IRs are closely related to iGluRs [29, 30]. A total of 45 and 50 IRs (including 6 and 8 pseudogenes, respectively) and 12 and 14 iGluRs (1 pseudogene in each) were identified in *H. phaleratus* and *H. cichorii*, respectively (Table 1). IR25a and IR8a are considered coreceptor IRs, which are highly conserved in beetles, and a 1:1 orthologue was present in our ML tree (Fig. 3). The conserved antennal IRs, including IR75, IR8, IR25 IR40a, IR21a, IR68a, IR60a, IR41a, IR76b and IR93a, were identified in blister beetles (Fig. 3 and Table 2). Seven of these ten clades contained only one copy in both blister beetle species, and five were present as 1:1 orthologues in all the analysed beetles (Fig. 3 and Table 2). Notably, a total of 10 (including one pseudogene) and 13 (including three pseudogenes) copies of IR41a were annotated in *H. phaleratus* and *H. cichorii*, respectively. However, the corresponding IRs in other species had no more than 3 copies (Fig. 3 and Table 2). IR41a was clearly expanded in blister beetles compared to other beetles. IR41a is not a unique example; for example, IR75 was expanded in blister beetle species, with 8 (including one pseudogene) and 13 (including three pseudogenes) copies in these two beetles, and the gene number of this clade was more variable than that of the other antennal IRs (Fig. 3 and Table 2). The remaining putative divergent IRs (18 and 16 in *H. phaleratus* and *H. cichorii*, respectively) almost all appeared as pairs in the phylogenetic tree. Two copies of IR100 were also identified in both species. The IRs showed high conservation between these two beetles. A total of 39 pairs of orthologous IRs were found between the two species, and 2 pairs of orthologues showed evidence of positive selection, namely, IR41a2–1 and IR112 (Table 1, Fig. 3 and Additional file 3).

**Table 2:** Number of Antennal IRs for the Seven Beetles. Major *Hycleus*-specific expansion of ‘IR41a’ is evident. ‘IR75’ expansion is observed in both the *Hycleus* genus and bark beetle. * One positively selected orthologue pair was identified in blister beetles

	*H. phaleratus*	*H. cichorii*	*T. castaneum* [31]	*D. ponderosae* [28]	*L. decemlineata* [31]	*A. planipennis* [28]	*A. glabripennis* [32]
*IR25a*	1	1	1	1	1	1	1
*IR8a*	1	1	1	1	1	1	1
**IR75**	**8**	**13**	**5**	**11**	**6**	**4**	**8**
IR40a	1	1	1	1	1	1	1
*R21a*	2	1	1	1	1	1	1
*IR68a*	1	1	1	1	1	1	1
IR41a*	**10**	**13**	**3**	**2**	**1**	**1**	**1**
*IR76b*	1	1	1	1	1	2	1
*IR93a*	1	1	1	1	1	1	1
*IR60a*	1	1	1	1	1	1	1
**Total**	**27**	**34**	**16**	**21**	**15**	**14**	**17**

**Fig. 4:**
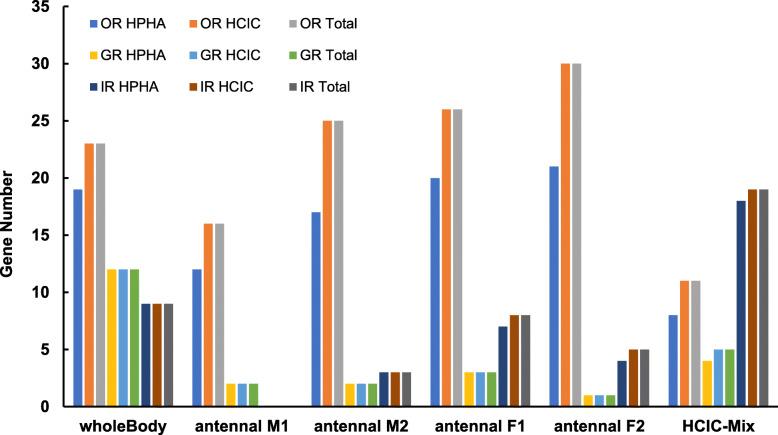
Evaluation of the Completeness of Annotation in the Genome using RNA-seq De Novo Assembly. Each sample includes nine bars: 1–3 are data for ORs, 4–6 are data for GRs, and 7–9 are data for IRs. The putative proteins identified via RNA-seq were also identified in the *H. phaleratus* genome (HPHA) and *H. cichorii* genome (HCIC), and the total number was identified by RNA-seq (Total). The first five samples were from *H. phaleratus* tissue (one whole-body sample and four antennal samples), and the last sample was assembled from a mixture of three whole-body samples of *H. cichorii* (see methods)

Chemosensory receptors are often present as tandem repeat clusters in the genome and are therefore more difficult to assemble than other genomic regions, which may represent the largest obstacle to annotating all members of such gene families. The current genomes of these blister beetles were assembled from next-generation sequencing (NGS) data, and the scaffold N50 length was only 79 kb in *H. cichorii* and 56 kb in *H. phaleratus*; moreover, Benchmarking Universal Single-Copy Orthologs (BUSCO) evaluation showed ~ 92% complete BUSCO in both species [33]. To verify whether some putative receptors were not assembled in the genome, we de novo assembled RNA-seq data and mapped these sequences onto the known corresponding protein and genome sequences. The results showed that all receptor genes identified via RNA-seq were also annotated in the *H. cichorii* genome (Fig. 4 and Table 1). Few GRs and IRs were missed in the *H. phaleratus* genome, while ~ 17.4–30.0% of the ORs identified via RNA-seq were missing from this genome (Fig. 4 and Table 1). In *H. cichorii*, all receptor genes identified via RNA-seq were annotated in the genome (Fig. 4), which may be beneficial for evolutionary analyses of these three gene families in blister beetle within Coleoptera.

## Discussion

We first identified the chemosensory receptor families at the whole-genome level in a pair of sister beetle species (Coleoptera: Meloidae) and performed an evolutionary analysis of chemosensory receptors within Coleoptera groups. In Coleoptera, surprisingly few studies have examined how sister taxa differ in chemosensory gene evolution, although two leaf beetles (*Pyrrhalta aenescens* and *Pyrrhalta maculicollis*) [6] and two distinct populations of the leaf beetle *C. lapponica* [7] have been partially assessed via RNA-seq. Hence, our study facilitates the analysis of chemosensory evolution among beetles, and we can now begin to discuss the question of how blister beetle diversification relates to the differentiation of these crucial gene families.

ORs are one of the key bridges between animals and their surroundings and play roles in the sensing of food, predators and potential mates. The OR family is the most widely studied among chemosensory receptors. Based on previous studies [27], nine groups of beetle ORs were identified in this study, and a similar pattern was observed in our phylogenetic tree. For example, most ORs of *D. ponderosae* and *A. planipennis* were classified in Groups 7 and 2B, respectively, while Groups 4, 5B and 6 contained very few beetle ORs (Fig. 1). Notably, two expanded clades of ORs were identified based on our phylogenetic tree (red arcs in Groups 5A and 3; Fig. 1), and more than 1/2 of the blister beetle ORs were included. Based on the phylogenetic tree, the divergence in these two clades is notably low, especially in Group 5A, indicating that these ORs present similar sequences and emerged in a relatively short time. Moreover, the selection signals of 8 pairs of ORs were detected, and 5 pairs were from these two expanded clades, while 4 pairs and 1 pair were from Groups 5A and 3, respectively. These data suggest that the ORs in these expanded clades may have emerged along with the differentiation of both blister beetles, with speciation occurring approximately 23 million years ago [33].

Up to 67% more ORs were identified in *H. cichorii* than in its sister species (149 vs 89 ORs), and nearly all of these additional *H. cichorii* ORs were clustered in two expanded clades (Groups 5A and 3). However, the ORs in the other clades showed nearly 1:1 orthologues in both blister beetles. On the other hand, our evaluation using transcriptome data showed that ~ 17.4–30.0% of the ORs identified via RNA-seq were missing from the *H. phaleratus* genome (Fig. 4 and Table 1). This finding suggests that some members in these expanded clades were not present in the genome assembly, which is the main reason why fewer genes were identified in *H. phaleratus* than in its sibling beetle species. In *H. cichorii*, the ORs identified via RNA-seq were annotated in its genome (Fig. 4), making for more robust evolutionary analyses within Coleoptera. Additionally, compared to another recent report, a higher proportion of pseudogenes was annotated in the ORs (20.1 and 22.5%), but the proportion was in the normal range since the value was close to that for the red flour beetle OR (23.2% pseudogenes) [34].

GRs are divided into four groups based on the type of contact material, namely, CO_2_, sugar, and fructose receptors and putative bitter receptors. We identified three, eight and three pairs of CO_2_, sugar and fructose receptors, respectively, in both blister beetle species (Fig. 2). These groups are consistently highly conserved in insects, while the putative bitter receptors consistently show high divergence. However, the putative bitter GRs also exhibited good conservation among members of the *Hycleus* genus, with more than two-thirds of the *H. phaleratus* bitter GRs existing in 1:1 orthologue pairs. Similar to the ORs, two recently expanded clades were identified in the bitter GRs, with one gene showing evidence of positive selection. These data suggest that the GRs in these expanded clades may also be linked to the differentiation of both blister beetles. Although the gene number of putative bitter receptors is slightly greater in *H. cichorii* than in *H. phaleratus* (87 vs 72), this difference may be related to the difference in genome quality, with the scaffold N50 at 79 kb in *H. cichorii* and 56 kb in *H. phaleratus* [33]. Moreover, nearly all the additional bitter GRs of *H. cichorii* were clustered in these expanded clades.

IRs are also membrane proteins that are closely related to iGluRs [29, 30]. We identified 45 and 50 IRs in *H. phaleratus* and *H. cichorii*, respectively. IRs can be divided into two groups: antennal IRs, which are consistently conserved among insect orders and function in olfaction in insects, and divergent IRs, which are highly divergent and expressed in peripheral and internal gustatory neurons, which indicates their involvement in taste and food assessment [29]. Ten clades of antennal IRs were identified in both blister beetles, and five of these subfamilies were conserved with a 1:1 orthologue in all seven beetles (Fig. 3 and Table 2). However, IR41a (10 and 13 in blister beetles vs 1–3 in others) was evidently expanded compared to the others (Fig. 3 and Table 2) and had the largest number identified in beetles to date. Based on the expansion reported in *D. ponderosae* [28], the expanded IR75 (8 and 13 in blister beetles and 11 in *D. ponderosae* [28] vs 4–8 in others) was also found in blister beetles. These data suggest that IR41a and IR75 underwent expansion in the *Hycleus* genus, which could have introduced new characteristics in blister beetles. One IR41a member (IR41a2–1) showed evidence of positive selection. To some degree, its expansion was accompanied by blister beetle speciation. We also identified IR60a (one copy in each beetle) in beetles based on our phylogenetic tree; this gene was misnamed in other beetles except for *D. ponderosae* and *A. planipennis*.

### Candidate chemosensory receptors linked to speciation

Divergent chemosensory receptors linked to differences in the sensory tuning of sister species represent a response to divergent selection of chemosensory traits [1]; thus, these chemosensory genes may be associated with speciation and directly or indirectly reflect the ability to adapt to the environment. For instance, a comparison of the gene duplication of chemosensory gene families between the sister beetle species suggested some candidate genes that might be associated with the ability to adapt to changing environments. In the present study, two evident expansions of OR clades were identified in blister beetles. Five of eight OR pairs showing evidence of positive selection were included in these expanded clades. Similar to ORs, two recently expanded bitter GR clades and one that includes a member with signs of positive selection were identified. Moreover, IR41a was expanded specifically in both blister beetles, and one IR41a protein showed signs of positive selection. These data suggest that these ORs, GRs and IRs may be linked to reproductive isolation between the species. Unfortunately, some receptors of *H. phaleratus* were missing from the genome, and these should be identified and analysed in the future. On the other hand, we identified 13 pairs of orthologues that showed signs of positive selection, i.e., 8 ORs, 3 GRs and 2 IRs. Seven of these pairs also appeared in the cluster of gene duplication, i.e., 5 ORs, 1 GR (GR34) and one IR41a (Additional file 3). These two beetles have largely overlapping sympatric ranges in Southwest China. Based on our field survey experience, the *H. phaleratus* population has declined in recent years due to the destruction of its habitats by human activity, including capture by humans. In contrast, the *H. cichorii* population has not obviously declined in a similar manner. These gene duplications and amino acid variations may facilitate greater adaptation to environmental changes in *H. cichorii* than in *H. phaleratus*. Other methods, such as changes in gene expression, should be considered in future studies.

## Conclusions

In the present study, we first performed comprehensive whole-genome identification of chemosensory receptor gene families in a pair of sister beetles (Meloidae: *Hycleus*). Several evolutionary events of blister beetles were revealed. For example, two expanded clades with positively selected proteins were identified in both ORs and bitter GRs, and two evident expansions of IR41a (blister beetle specific) and IR75 (*Hycleus* genus and *D. ponderosae*) were detected. This study will provide an important platform for future functional characterization of chemoreceptors and for uncovering the evolutionary history of blister beetles.

## Methods

### Identification of chemosensory receptors

The *H. cichorii* and *H. phaleratus* genome sequences were obtained from the NCBI database (BioProject accession number PRJNA390850) [33]. We identified OR, GR, and IR genes using the following steps. First, we constructed a protein reference database of each family using published proteins from *Tribolium castaneum*, *D. ponderosae*, *Anoplophora glabripennis*, *A. planipennis* and *Leptinotarsa decemlineata* [27–29, 31, 32, 35]. Second, tBLASTn (version 2.2.25) was used to search the exons in both genomes with an e-value cut-off of 1e-5. Third, we first linked the alignment hits into candidate gene loci exon by exon with an in-house Perl script. We then extracted genomic sequences of candidate loci together with 2-kb flanking sequences and used GeneWise [36] to determine the gene models. Next, putative proteins were manually compared with other genes of the same family and RNA-seq data (NCBI SRA database, SRA accession number SRR5408725, SRR5408726, SRR5757329, SRR5757330, SRR9702113, SRR9702114, SRR9702115, and SRR9702116 for *H. phaleratus* and SRR4436644, SRR4436645, and SRR1996329 for *H. cichorii*), and only the best gene model/splice isoform with the highest score provided by GeneWise was retained. For incomplete genes, putative proteins with at least half the length of the reference protein and a size larger than 190 amino acids were retained. Genes with premature stop codons or frameshifts were defined as pseudogenes. The premature stop codons were replaced by X in the translated sequence. Finally, we assessed functional annotations in public protein databases (NCBI nonredundant protein and Swiss-Prot) using BLASTp and removed candidate genes with inconsistent family annotations. To avoid missing some putative genes, we performed two rounds of identification using the above pipeline. In the second round, the putative proteins identified in the first round were also included as queries for tBLASTn and GeneWise.

To verify whether some chemosensory receptor genes were missing in the annotation process and in the genome, we de novo assembled RNA-seq data and counted the number of candidates. The RNA-seq data for *H. phaleratus* (whole body and antennae) were obtained from the NCBI SRA database (SRA accession number SRR5408725, SRR5408726, SRR5757329, SRR5757330, SRR9702113, SRR9702114, SRR9702115, and SRR9702116), and the data for *H. cichorii* were also obtained from the NCBI SRA database (SRA accession numbers: SRR4436644, SRR4436645, and SRR1996329). After removing adaptor commination and low-quality reads, Trinity (version v2.0.6; http://trinityrnaseq.sourceforge.net/) [37] was applied with the following parameters: group_pairs_distance = 300; min_contig_length = 150; min_kmer_cov = 3; and min_glue = 3. Then, the assembled sequences were mapped onto the known reference protein (the same proteins as in the identification step) and genome sequences with BLAT [38], and the candidates were counted, the homologous regions of which contained at least 120 (ORs and GRs) or 190 (IRs) amino acids.

### Phylogenetic trees

To identify orthologous pairs between the two sister species and analyse the relationships of *Hycleus* chemosensory receptors with those of other insects, ML trees for each family were constructed using the amino acid sequences derived from the putative CDSs and the published proteins of other species, including *T. castaneum*, *D. ponderosae*, *A. planipennis* and *L. decemlineata* (Additional file 4) [28, 31, 32, 35]. First, multiple-sequence alignment was performed by MAFFT (version 7.407) [39] using the amino acid sequences corresponding to the chemosensory receptors of these species. Then, RAxML (v8.2.12) [40] was used to construct phylogenetic trees of the chemosensory receptors with the best models (JTT for IRs, JTT + G + F for ORs and GRs), and node support was assessed using a bootstrap procedure based on 200 replicates. The phylogenetic trees were visualized with EvolView (http://evolgenius.info/evolview) [41]. To facilitate a more robust phylogenetic analysis, partial ORs with fewer than 230 amino acids were excluded from the phylogenetic analysis. Due to the highly divergent nature of GRs, amino acid sequences with fewer than 250 amino acids were also excluded [28]. The OR tree was rooted with the conserved lineage of odorant receptor coreceptor (Orco) proteins, the GR tree was rooted with the conserved lineage of putative sugar receptors, and the IR tree was rooted with the conserved lineage of putative IR8a/25a proteins.

### Selection analysis

According to the phylogenetic trees, we selected the orthologous gene pairs between these two *Hycleus* species for selection analysis. The paired orthologues of the protein sequences were aligned using MAFFT (version 7.407) [39]. Then, the alignment information of the protein sequences was used as a guide for nucleic acid sequence alignment. Finally, analyses of nonsynonymous (KA) and synonymous (KS) substitutions were performed by codeml in the PAML package (v4.9) [42] using the site model (M8 [beta and ω > 1] vs M7 [beta]). To test whether each site in an orthologue pair underwent significant selection, the likelihood ratio was estimated based on the M8 model and M7 model. The LRT statistic uses the formula Δ = 2(ln(M8)-ln(M7)), with Δ approximating a chi-square distribution with one degree of freedom [26]. The putative selected genes had at least one selected site and passed the chi-square test using a *P*-value cut-off of 0.01. All the selected sites had to pass both test methods (Bayes empirical Bayes [BEB] and naive empirical Bayes [NEB], provided by codeml software) using a probability cut-off of 0.95, and the quality of the alignment was manually assessed.

## Supplementary information

**Additional file 1.** Amino acid and nucleotide sequences of ORs, IRs and GRs identified in this work.

**Additional file 2.** Gene location information (gff).

**Additional file 3.** All positively selected gene pairs between the two blister beetle species.

**Additional file 4.** The protein set used in phylogenetic tree analysis.

## Data Availability

All data generated by this study are included in this published article and its supplementary information file 1–3. The proteins of *T. castaneum*, *D. ponderosae*, *A. glabripennis*, *A. planipennis*, and *L. decemlineata* were retrieved from the previous publications, used as BLAST query [27–29, 31, 32, 35] and for phylogenetic tree were summarized in Additional file 4. The reference genome sequences of *H. cichorii* and *H. phaleratus* were obtained from the NCBI database (BioProject accession number PRJNA390850). The RNA-seq data were used for annotation and check from NCBI SRA database (SRA accession number SRR5408725, SRR5408726, SRR5757329, SRR5757330, SRR9702113, SRR9702114, SRR9702115, and SRR9702116 for *H. phaleratus*; and the SRR4436644, SRR4436645, and SRR1996329 for *H. cichorii*).

## Abbreviations

GR: Gustatory receptor
OR: Odorant receptor
IR: Ionotropic receptor
iGluR: Ionotropic glutamate receptor
ML: Maximum likelihood
CDS: Coding sequence
NGS: Next-generation sequencing
BUSCO: Benchmarking Universal Single-Copy Orthologs
